# Supplementary figures and images for: An Atlas of Continuous Adaptive Evolution in Endemic Human Viruses
Source: Cell Host Microbe. Author manuscript; Available in PMC 2025 Jun 2. (PMC12129310; doi:10.1016/j.chom.2023.09.012)

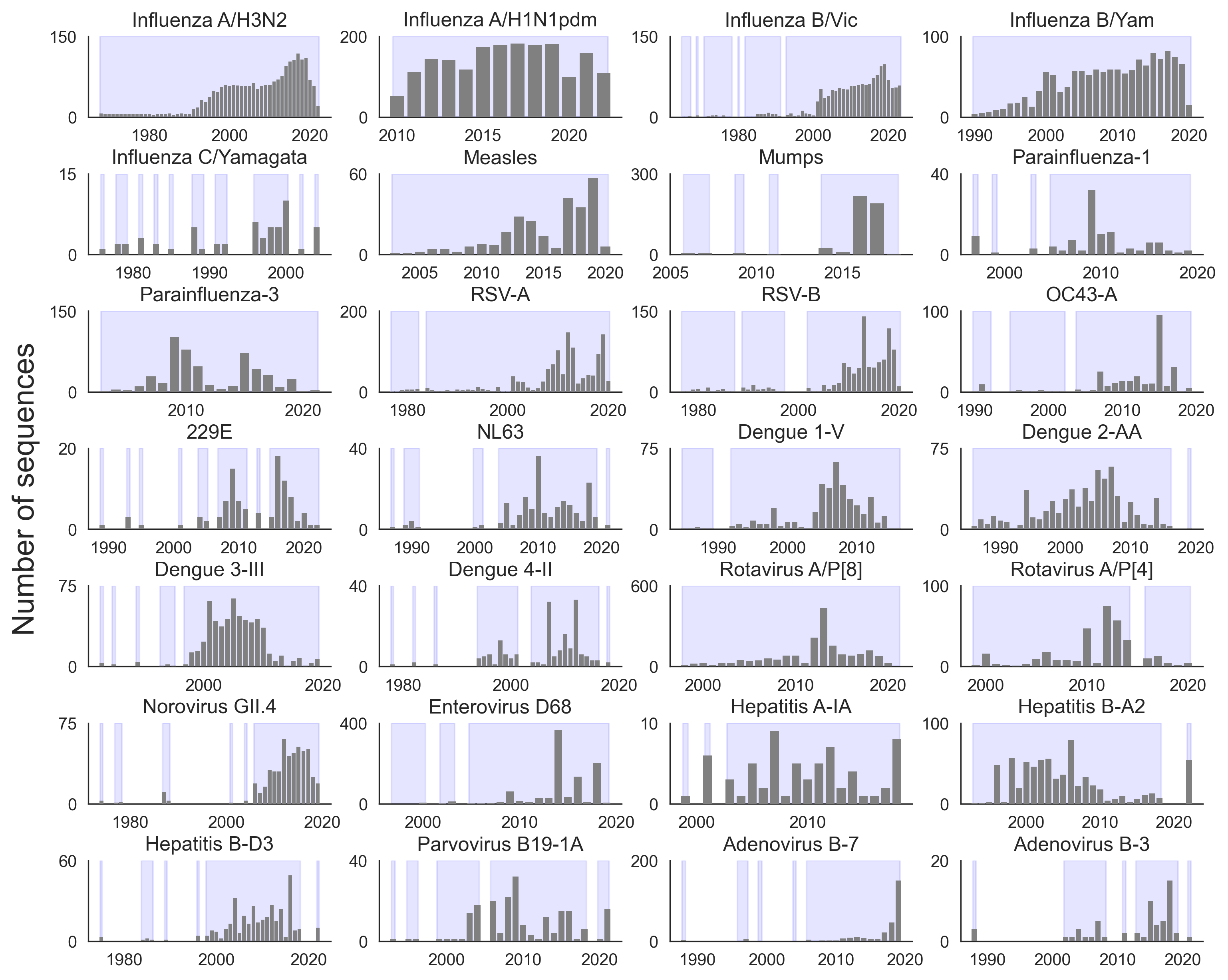

Supplement: Figure S2 [file NIHMS2080518-supplement-Figure_S2.png]

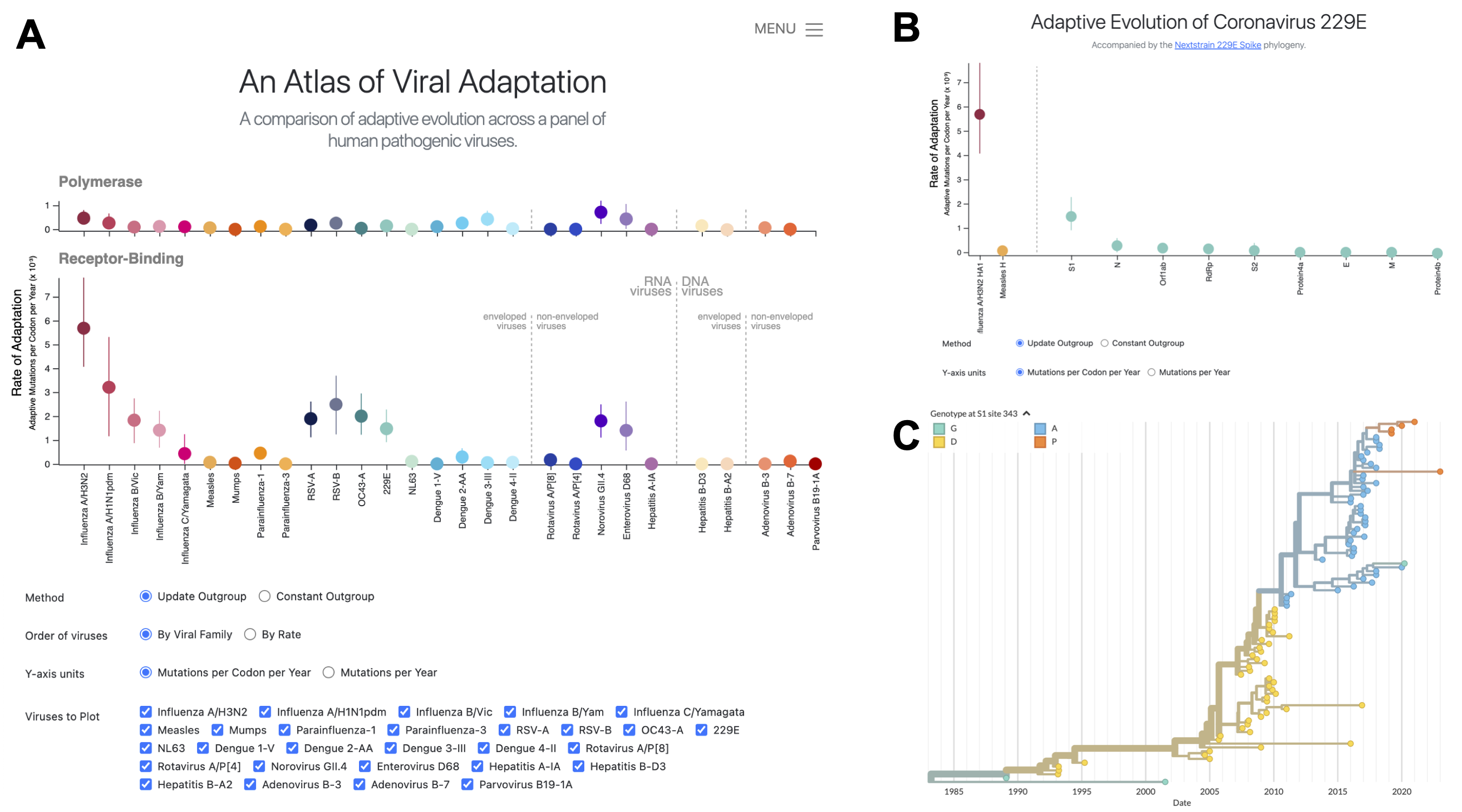

Supplement: Figure S1 [file NIHMS2080518-supplement-Figure_S1.png]
